# Beyond the stigma: a community-based mixed methods hybrid photovoice–appreciative inquiry protocol to explore and enhance engagement of young men in mental health research

**DOI:** 10.1136/bmjopen-2025-115182

**Published:** 2026-07-13

**Authors:** David Francis Hunt, George Mycock, Tobit Emmens, Matt Young, Steph Scott

**Affiliations:** 1 Psychology, University of Exeter, Exeter, UK; 2 Devon Partnership NHS Trust, Exeter, UK; 3 University of Exeter, Exeter, UK; 4 Who Needs Instructions CIC, Exeter, UK; 5 Population Health Sciences Institute, Newcastle University Faculty of Medical Sciences, Newcastle upon Tyne, UK

**Keywords:** MENTAL HEALTH, Community Participation, Health Services, Health Equity

## Abstract

**Abstract:**

**Introduction:**

Young men’s mental health is a continuing concern but remains underrepresented in both mental health services and research. Engaging young men in mental health research has proven difficult, but creative, strength-based and participatory methods may help overcome barriers and enhance understanding of how best to involve them. This study aims to explore young men’s perceptions of how healthcare and academic settings can better engage them in mental health research, and to examine whether participatory, strength-based approaches themselves foster greater engagement.

**Methods and analysis:**

This convergent mixed-methods participatory-informed study integrates photovoice with appreciative inquiry (AI) within a four-phase AI cycle: Discovery (photovoice-led exploration of ‘what works’ in engaging with research), Dream (co-imagining desirable engagement experiences), Design (co-creating practical engagement resources and practices) and Deploy (planning how to test and refine these practices). Participants will be young men aged approximately 18–25 years, recruited through community organisations and primary-care-adjacent networks.

Data will include photographs with captions and written captions (Discovery), workshop transcriptions (Dream/Design/Deploy). Qualitative data will undergo reflexive thematic analysis, incorporating participatory sense-making through member reflections. Quantitative data will analyse pre–post changes on engagement and empowerment measures, which will be summarised with means, medians and bias-corrected bootstrapped 95% CIs, with exploratory non-parametric tests where appropriate. Feasibility and acceptability will be assessed through recruitment, retention, data completeness and satisfaction metrics. Adverse events and distress responses will be monitored and reported.

**Ethics and dissemination:**

Ethical approval has been granted by the University of Exeter Ethics Committee (Ref 10981599). Safeguards include staged written consent, image ownership and usage agreements, anonymisation and a distress and safeguarding protocol with supported signposting. All participants will provide informed consent, and data will be managed in accordance with GDPR and university information governance policies. Findings will be shared with participants and community partners, published in peer-reviewed outlets and developed into a replicable engagement toolkit for wider use.

**STRENGTHS AND LIMITATIONS OF THIS STUDY**

- Introduces a hybrid participatory methodology (photovoice+appreciative inquiry) that treats engagement as both process and outcome.
- Embeds co-analysis and co-design to enhance practical relevance for research teams and services seeking to reach young men.
- Combines pre-/post-engagement metrics with qualitative and visual data to assess feasibility, acceptability and potential impact on participation.
- Findings will be context-bound; transferability rather than generalisability is the goal.
- The study focuses on methodological and system-level engagement, not on clinical outcomes.

## Introduction

 Young men (YM) face significant challenges in accessing mental health support and remain underrepresented and underserved in both services and research. They account for around three-quarters of youth suicides,^1 2^ and one in five experience mental health difficulties (3). High rates of substance misuse and a marked rise in the number of YM not engaged in education or employment further compound these difficulties.^3^,^5^ Despite this burden, YM and men more broadly are less likely to access services or participate in research, contributing to poorer outcomes and less effective treatments and engagement strategies.^4 6 7^

Barriers to engagement are multifaceted for YM. They are less likely than other groups to seek help or take part in research due to low mental health literacy, adherence to masculine norms of self-reliance, stigma (self, structural and societal) and emotional restriction.^7^,^12^ A local NHS England Research Engagement Network project highlighted additional systemic barriers, including service opening hours, the predominance of female gatekeepers (eg, reception and administrative staff) and fears of public humiliation.^13^ Attributing non-participation solely to individual behaviour risks ‘victim-blaming’ and overlooks these wider structural constraints.

As a result, the perspectives of YM remain absent from mental health research, limiting understanding of their unique needs and perpetuating inequities in support.^9 12^ However, it is important to recognise that YM are not a homogeneous group, and their needs vary across backgrounds, identities and contexts. Distrust of services, reluctance to appear vulnerable and perceptions that interventions do not reflect their concerns further contribute to disengagement. To design effective and accessible interventions, it is essential to explore innovative approaches that enable YM to participate on their own terms.

Emerging evidence suggests participatory and creative methods offer promise in addressing these barriers.^9 10 13^ Photovoice provides a creative medium that reduces stigma, allows participants (lived experience partners) to control their narratives and uses visual metaphors to support expression of complex ideas.^14^,^16^ This approach can leverage the technological fluency of YM to enhance literacy and confidence in discussing mental health.^10 12 16^ Complementarily, appreciative inquiry (AppInq) emphasises solutions and strengths, helping YM to build agency, reframe masculinity norms and identify actionable outcomes in ways that build trust and enhance accessibility.^16 17^ Integrating these approaches may offer a novel, strength-based route to engagement.

This study addresses the underserving of YM in mental health research through two purposes: (1) to explore the perspectives of YM of what facilitates the participation of YM in mental health research and (2) to pilot the combination of photovoice and AI methodologies with YM. This strength-based method offers a potentially accessible way for YM to share their perspectives and generate strategies to improve their engagement in mental health research.

### Aim and research questions

This project aims to develop and test innovative approaches to engaging YM in mental health research by combining photovoice and AI, with the goal of addressing barriers to participation and ensuring the perspectives of YM inform mental health research and subsequent service improvements. The related research questions (RQs) are as follows:

1. What are the factors that enable YM to engage meaningfully in mental health research?
2. What insights from YM highlight ways of designing participatory mental health research with this population?
3. How do YM experience and evaluate combined photovoice–AI approach as a method for engaging themselves and other YM in mental health research?
4. How do YM experience the combined photovoice–AI approach, and what changes in engagement and empowerment-related constructs are observed across participation?

## Methods and analysis

### Study design

This study will pilot a convergent mixed-methods participatory-informed design in which qualitative and quantitative data are collected alongside one another to address related but distinct aspects of engagement in mental health research. Participant recruitment is planned between November 2025 and January 2026, with study procedures and data collection scheduled from late January to late February 2026. The qualitative strand forms the primary component of the study and explores perspectives of YM regarding how mental health research can better engage their cohort. The quantitative strand complements this by examining the feasibility, acceptability and potential influence of the combined photovoice–AppInq approach on empowerment and engagement-related constructs.

Qualitative and quantitative strands will be analysed separately and brought together, if appropriate, during interpretation to explore how the experiences of participants of the method relate to the patterns observed in the quantitative measures. Integration will primarily serve a complementary and contextualising purpose rather than seeking direct equivalence between both strands of findings.

Photovoice supports expression, agency and narrative control, while AppInq structures these insights into forward-looking, solution-focused discussions. Integrating the two enables a movement from representation to reflection and collaborative co-design. The qualitative strand forms the core of the study and will take place in the AppInq workshop, where participants will use their photovoice images as elicitation tools during focus group discussions. These discussions will follow a solution-focused and strength-based AppInq process that supports YM to explore their experiences and to collaboratively develop strategies for improving engagement in mental health research.

**Figure 1:**
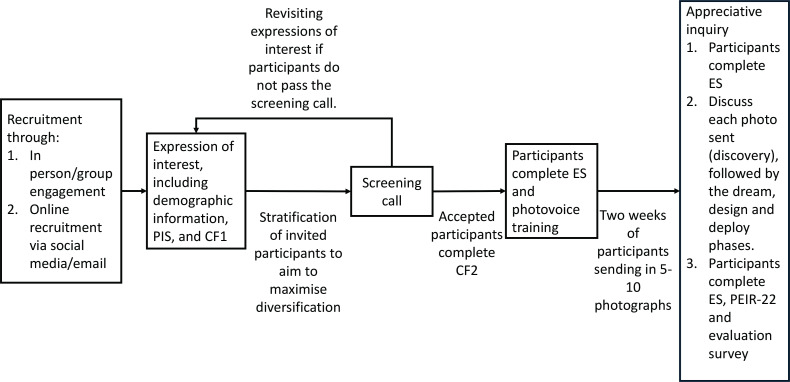
An illustrative map of the present study design. CF, consent form; ES, Empowerment Scale; PEIRS-22, shortened Patient Engagement in Research Scale; PIS, participant information sheet.

The complementary quantitative component will explore patterns of change in empowerment and engagement-related constructs across three time points (baseline, pre-AppInq workshop, post-AppInq workshop) as outlined below to test whether the approach increases participant engagement. Feasibility and acceptability will be assessed through recruitment and retention rates, fidelity to the AppInq workshop, completeness of data and brief satisfaction items. Adverse events and distress procedures will be monitored and reported throughout the study. Distress will be managed in line with the risk protocol adopted from the Mood Disorders Centre at the University of Exeter. An overview of the approach is illustrated in (figure 1).

### Recruitment and sampling

This study aims to recruit approximately 15 YM aged 18–25, with self-identified experience with mental health concerns, from diverse backgrounds (eg, ethnicities, socioeconomic statuses and rural/urban settings), using a purposive sampling approach. The number balances sample diversity with the practical feasibility of a pilot study. Research reporting recruitment strategies for men suggests targeting in-person and online spaces where the target population are already located.^18^,^20^ Online recruitment strategies are commonplace in modern research, especially post-COVID-19 pandemic, where more members of the public shifted to online work.^21^ Given that Reddit’s user base is predominantly male and widely used by those aged 18–25,^22 23^ local subreddits such as r/Exeter and r/ExeterUniversity will likely be used to reach YM in the community. As this study looks to explore the recruitment of YM for mental health research specifically, we hope to recruit YM who have experience with mental health concerns, who may or may not have sought/accessed support for their mental health. To reach YM with lived experience of mental health concerns, recruitment will also draw on existing stakeholder networks within the research team, including the Devon Partnership NHS Trust Equality, Diversity and Inclusion group, the men’s mental health community Who Needs Instructions, the University of Exeter and other local organisations.

Recruitment materials will link to an online expression of interest form containing study information, consent and anonymous demographic questions. Participants will create a participant identification code that will allow the researchers to link the anonymous expression of interest form to a separate survey which will collect contact details. These contact details will be stored separately to protect the confidentiality of participants.

A purposive sampling approach will be used to recruit up to 15 YM from diverse backgrounds (eg, ethnicity, sexual orientation, socioeconomic status, rural/urban location and help-seeking history). Given the small sample size and pilot nature of the study, diversity is sought not to achieve representativeness but to assess the feasibility and inclusivity of the method across YM with varied backgrounds and lived experiences.^8 24 25^ Capturing variation across these characteristics will support a preliminary assessment of inclusivity and feasibility.^26^

Selected participants will be invited to a brief screening video call to confirm eligibility and authenticity, following guidance on identifying false participants.^27^ During the call, the study purpose and payment schedule will be clarified, and eligible individuals will be invited to the in-person photovoice training session after completing an additional informed consent form. The screening call is used to confirm eligibility, ensure that individuals understand the study requirements and minimise fraudulent participation—an increasingly common issue in online research. This process does not involve any assessment of personal sincerity or ‘authenticity’.

### Patient and public involvement

Two members of the research team have relevant lived experience, and an expert by experience involved in the development of the study to shape the design, approach and overall framing of engagement with YM in mental health research. Their input informed refinement of the study aims, recruitment approach, workshop structure and the acceptability of proposed methods. This early involvement helped ensure that the study design was grounded in lived experience perspectives regarding barriers to participation and meaningful engagement.

Two experts by experience will also support piloting the study procedures to ensure the methods are appropriate and accessible. Their feedback on feasibility informed the development of recruitment materials and strategies. Our experts by experience and participants will also be involved in the dissemination of findings through the Exeter-based exhibition and potential co-authored outputs reflecting on experiences of the participatory process.

## Data collection

### Quantitative measures

To address RQ4, measures of engagement and empowerment will be used to assess whether participation in the intervention increases these constructs.

#### Engagement

The Patient Engagement in Research Scale-Short Form (PEIRS-22^28^) will be administered post-intervention to assess the perceived quality and depth of the engagement of participants. This is a 22-item instrument with a good level of validation.^28^,^30^ This will be a post-measure only as measuring engagement is only logical after participation.

#### Empowerment

Empowerment is included as it reflects constructs central to participatory approaches and is highly relevant for understanding whether YM experience increased confidence and ownership through the combined method. Changes in the sense of empowerment of the participants will be assessed using the Empowerment Scale,^31^ a 28-item instrument across five subscales: self-efficacy, power/powerlessness, community activism, optimism and control over future and righteous anger.^32^ The ES will be administered at three stages: baseline (photovoice training), pre-AppInq workshop and post-AppInq workshop to explore changes in empowerment alongside qualitative reflections.^33^

### Qualitative methods

To provide answers to RQ1, 2 and 3, the present study will undertake a two-phase qualitative investigation integrating a creative method (photovoice) and a strength-based discussion framework (AppInq).

### Photovoice/appreciative inquiry

For this study, photovoice^34^ methods are integrated with AppInq by using photovoice to enrich AppInq-informed discussions with participants (described below). Through this integration of methods, the study aims to target the culmination and development of ideas to facilitate the involvement of YM with mental health research.^35^ Indeed, a review by Catalani and Minkler^36^ reports that, across various photovoice studies, engaging in critical discussions with participants as part of the photovoice method often led to valuable data collection and facilitated participants’ empowerment.

The photovoice and AppInq components will be delivered across two in-person meetings. The first will be a 2-hour photovoice training session and the second will be a 4-hour AppInq workshop. The training session is the recommended first step in photovoice and will explain the aims of the project, understanding the tasks outlined, how photographs and narratives will be used in the study outputs and the ethical requirements that apply to image content. The training will offer guidelines that aim to expand rather than limit how participants’ view and represent community assets.^34^

In addition to introducing the photovoice process and ethical guidance, the training session will function as an initial relational and participatory space in which the researchers and participants discuss why this research matters and how this might be represented through photography. These discussions are intended to support relationship-building, establish a psychologically safe and collaborative environment and inform how participants approach the subsequent photovoice activities.

The session will also contribute to the feasibility assessment by showing whether the photovoice tasks are accessible and usable for YM with varying levels of prior experience or familiarity with creative approaches, with the training providing a common foundation. As part of the wider feasibility assessment, we will collect baseline measures of engagement and work with YM to understand what forms of support or adjustments may be needed to enhance recruitment and sustained involvement in mental health research. Technical instruction will be kept to a minimum to avoid restricting creativity. Participants will be encouraged to produce a varied set of images illustrating how mental health research could better engage YM, with each image accompanied by a brief narrative.

Following the training meeting, participants will be instructed to take photographs over the 2 weeks, with the aim to submit 5–10 photos and narratives each prior to the AppInq discussion meeting. During the 2 weeks, researchers will remain in contact via email to encourage participants and offer the opportunity for participants to ask questions. Participants will submit their photos via email to a dedicated email address.

### Using the 4D model of AppInq

After the 2 weeks of photovoice, the participants will join the research team for a 4 hour AppInq workshop, using the 4D model—*Discover, Dream, Design and Deploy*—to structure discussions.^37 38^ This framework supports a strength-based exploration of how YM can be more effectively engaged in mental health research. The process begins with discussing what is elicited from the photographs in the Discovery stage, moves to considering how these insights can inform an ideal future for engagement in the Dream stage, identifies what needs to be developed in the Design stage and concludes with agreeing practical steps for taking these ideas forward in the Deploy stage. table 1 provides the interview schedule for each of the four stages during the workshop.

**Table 1:** Interview schedule

Stage of 4D model	Questions
Discovery (with photos)	Which photo did you choose, and what does it represent to you?
	How does this photo connect to your experiences of mental health or mental health research?
	Can you share a time when you (or someone you know) felt comfortable, supported or positive about engaging with mental health or research?
	What strengths or values in young men does this photo reflect that could help increase engagement?
Dream	Imagine a future where young men are actively and positively involved in mental health research—what would that look like?
	How would young men feel about being part of research in that future?
	What would make them excited, motivated or proud to take part?
Design (based on the themes rapidly identified from the Discovery/Dream phases)	What practical things could researchers or communities do to make young men feel more interested and included?
	What spaces, activities or approaches would feel most inviting and respectful?
	How should mental health research be communicated so it connects with young men?
Deploy	What is one small step we could take right now to start moving toward that dream?
	Who needs to be involved to make it happen?
	If you could give one piece of advice directly to researchers about engaging young men, what would it be?

### Data collection and analysis

Quantitative and qualitative data will be analysed separately and then brought together for interpretation and integration through triangulation so that the feasibility and early effects of the combined approach can be understood alongside participants’ lived experiences of the method.

### Quantitative analysis

Quantitative analyses will be conducted in Jamovi. Descriptive statistics (means, SD, medians and IQRs) will summarise participant characteristics, empowerment (Empowerment Scale) and engagement (PEIRS-22). Pre- and post-scores on the Empowerment Scale will be compared using estimation methods rather than null-hypothesis testing. Mean differences will be reported with 95% bias-corrected bootstrapped CIs (2000 resamples) and corresponding effect sizes (*Hedges’ g* or *Cohen’s dz*). Given the small sample, analyses will be exploratory and focused on patterns and feasibility rather than statistical significance. Where appropriate, non-parametric comparisons (eg, Wilcoxon signed-rank test) may be used to describe direction of change.

Individual trajectories will be shown using spaghetti plots to illustrate variation and direction of change. Group level patterns will be presented using estimation plots that display the raw data, mean change and bootstrapped CIs.

Given the small sample and the focus on this being a pilot study, quantitative analyses are included to assess feasibility, acceptability and sensitivity to change rather than to estimate population parameters. These data will help determine whether empowerment and engagement measures are appropriate for inclusion in a future larger study.

### Qualitative analysis

Qualitative data—including saved images, photovoice captions, audio reflections and AppInq discussions—will be managed and coded in NVivo using reflexive thematic analysis.^39 40^ Analysis will proceed through iterative familiarisation, coding, theme development and review, attending to both semantic and latent meanings. Visual data will be interpreted alongside narrative reflections, with attention to symbols, context and recurring ideas related to engagement and empowerment. This approach allows for collaborative discussion between the research team and participants. These methods also allow for a value-inclusive axiological stance to recognise the influence of researcher positionality, using reflexive notes throughout to document researcher perspectives and decision-making. Reflexive notes will be recorded after each session and during analysis meetings to document positionality, decision-making and emerging assumptions. These notes will inform team discussions and contribute to the transparency and rigour of the analytic process.

Participants will be provided with a summary of the preliminary themes via email and invited to comment to ensure interpretations reflect their perspectives. Their feedback will be documented and integrated into theme refinement.

Trustworthiness will be enhanced through multiple strategies aligned with qualitative rigour principles. Credibility will be supported through prolonged engagement across the training session, photovoice activities and workshop discussions, alongside member reflections on preliminary themes. Dependability and confirmability will be supported through reflexive field notes, collaborative analytic discussions within the research team and transparent documentation of analytic decision-making. Transferability will be supported through description of participants’ experiences, study context and methodological processes. Triangulation across visual, narrative, discussion and quantitative data sources will further support the depth and rigour of interpretations.

### Feasibility indicators

Process measures will be taken with a focus on the recruitment and retention rates for the study. These indicators will include:

- ≥70% of enrolled participants attend the photovoice training session.
- ≥80% of those who attend training submit at least one usable photo and narrative, and≥60% submit five or more images.
- ≥60% of those who attend training attend the AppInq workshop.
- ≥70% of workshop attendees complete the post-intervention measures with≥80% item completeness.
- ≥60% of enrolled participants complete all study components.

These thresholds are based on typical expectations for early-phase participatory pilot work and are intended to guide refinement of the approach rather than to act as formal progression criteria.

### Triangulation

Quantitative and qualitative data will be analysed separately and then brought together for interpretation. Integration will focus on how participants’ experiences and reflections regarding engagement relate to patterns observed in empowerment and engagement-related measures. Joint displays will be used to support comparison and contextualisation across data sources, allowing areas of complementarity to be explored. Divergence between qualitative and quantitative findings will be treated as potentially informative for understanding variation in how participants experience the combined photovoice–AI process.

## Ethics and dissemination

### Ethics

The study has received University of Exeter Ethics Approval (Ref 10981599), which underwent a triage process with the central ethics team and a peer review via the department-level review process. During photovoice and AppInq discussions, participants may reflect on personal or potentially distressing experiences. To support participants’ wellbeing, they will receive a debrief sheet listing national and local mental health support services (telephone, text and online). This will be provided at multiple stages—on invitation to the screening call, after photovoice training and before and after AppInq discussions.

Participants will also receive clear guidance on the ethical requirements for photovoice. They will be instructed not to photograph identifiable individuals or recognisable locations, and third-party consent will be required if such content is unavoidable. Images will be reviewed to ensure anonymity, including blurring faces where necessary. During photovoice training, participants are informed that submitted images may be anonymised in this way to protect privacy, and consent for these procedures is obtained in advance. Participants will provide specific consent for the use of anonymised images and narratives in publications and public dissemination and may withdraw any image prior to dissemination.

Due to the sensitive nature of the study and the lived experience of some team members, steps will be taken to support researcher well-being. The active researchers (GM and DFH) will hold regular debriefing meetings, with additional check-ins after any interviews that may be emotionally demanding. The wider team (MY, TE, SS) will meet periodically with the active researchers to provide further reflection and support. If any researcher becomes distressed, data collection may be paused, and the supervisory lead (DFH), who oversees the project and provides academic supervision, will review the situation. Researchers also have access to the university’s well-being and counselling services, which can be used confidentially at any time.

Participation is voluntary, and individuals may pause, skip questions or withdraw at any point. Reimbursement is offered on a pro-rated basis. Participants receive payment for attending the photovoice training session, and full payment is provided upon completion of the AppInq workshop. Facilitators are trained to recognise distress and will monitor verbal and non-verbal cues such as agitation, expressions of acute distress or indications of current risk. If these thresholds are met, the session will be paused and the participant will be checked in with, following the Mood Disorders Centre safeguarding protocol, including immediate support, referral to appropriate services and notification of the clinical supervisory support, who provides clinical oversight and risk-management guidance, where required. Researchers will keep reflexive field notes to document ethical considerations as they arise, and the strength-based, solution-focused design is expected to help mitigate distress by promoting agency, highlighting positive experiences and encouraging constructive discussion.

Data will be managed in accordance with GDPR and University of Exeter policies. Ethical approval has been granted by the University of Exeter Ethics Committee (Ref 10981599). The study follows the Standards for Reporting Qualitative Research to ensure transparency and rigour, and the Guidance for Reporting Involvement of Patients and the Public to document participatory and public-involvement processes.

### Dissemination

Findings will be disseminated through multiple channels to maximise reach and impact. Implementation ideas, photographs and narratives will be shared with *Devon Partnership NHS Foundation Trust*, the *Coalition for Men and Boys* and relevant men’s mental-health networks, as well as through social-media platforms. A public engagement event will be held in Exeter, inviting stakeholders from health, research and community sectors to discuss applications of the findings. Participants will also be invited to contribute to dissemination and interpretation activities where appropriate, including participation in the public engagement event, collaborative discussions regarding interpretation of findings and a potential co-authored output reflecting on experiences of the participatory process.

We anticipate submitting the full findings for publication to complement this protocol and will prepare a linked discussion paper exploring implications for participatory practice. The research team will also engage with the *Centre for Policy Research for Men and Boys* and contribute to the emerging *National Men’s Health Strategy*.

## Discussion

This study addresses a significant gap in mental health research by focusing on how to engage YM who remain underserved by current services and underrepresented in research. Existing studies have identified barriers to engagement but offer little guidance on how these barriers can be overcome. The proposed study introduces an innovative approach that brings together photovoice and AppInq to support YM to express their experiences and to contribute directly to practical strategies for improving engagement. This shift from description to collaborative problem solving represents a methodological advance for research with this population.

Publishing this protocol is important because the combined approach has not previously been tested with YM and requires careful assessment of feasibility and acceptability before wider use. Transparent reporting will support reproducibility and allow other researchers and practitioners to build on the design. Early dissemination is particularly important given the pressing need for methods that are acceptable, accessible and capable of involving YM from diverse backgrounds.

The study will provide initial insight into supporting YM engagement and create a foundation for refining participatory approaches that can be adapted for different groups. Strengthening engagement in research is a necessary first step towards improving the design of services and interventions that better meet their needs.

## Data Availability

No data are available.
