# Supplementary material for: Beyond the stigma: a community-based mixed methods hybrid photovoice–appreciative inquiry protocol to explore and enhance engagement of young men in mental health research
Source: BMJ Open. 2026 Jul 13;16(7):e115182. doi: 10.1136/bmjopen-2025-115182 (PMC13365781; doi:10.1136/bmjopen-2025-115182)
Supplement: Supplementary file 1 [file bmjopen-2025-115182.reviewer_comments.pdf]

PEER REVIEW HISTORY

BMJ Open publishes all reviews undertaken for accepted manuscripts. Reviewers are asked to complete a checklist review form and are provided with free text boxes to elaborate on their assessment. These free text comments are reproduced below.

ARTICLE DETAILS

Title (Provisional)

Beyond the Stigma: A Community-based Mixed Methods Hybrid Photovoice–Appreciative Inquiry Protocol to Explore and Enhance Engagement of Young Men in Mental Health Research

Authors

Hunt, David; Mycock, George; Emmens, Tobit; Young, Matt; Scott, Steph

---

VERSION 1 - REVIEW

Reviewer **1**

Name **Hai, Tasmia**

Affiliation **McGill University Department of Educational and Counselling Psychology**

Date **07-Mar-2026**

COI **None**

---

the protocol is well written

Reviewer **2**

Name **Ramsden, Vivian**

Affiliation **University of Saskatchewan, Department of Academic Family Medicine**

Date **14-May-2026**

COI **None**

The Investigators indicated that they were planning a convergent mixed-methods participatory study which describes the integration of photovoice with Appreciate Inquiry. It would be helpful to better understand the methods because the Investigators go on to focus on the fact that the study is being designed to address the fact that young men do not often participate in mental health research. Young men frequently need to be engaged in the co-

creation of the project if they are going to consider how best to address the barriers to participation which subsequently may lead to service improvements.

Given that this is a Protocol, it would be helpful for the Investigators to specifically frame how the qualitative (Photovoice and Appreciative Inquiry [AI]) and the quantitative (questionnaires at three points – baseline, pre-AI Workshop and post-AI Workshop) will increase engagement and confidence. A convergent mixed-methods study should answer the question, to what extent do the quantitative results and the qualitative findings converge. I highlight this because this is all supposed framed within the context of participatory research which I am struggling to identify clearly As Dr. Ann Macaulay has indicated over the years, the origins of participatory research were grounded in increasing social justice by promoting voice to disadvantaged populations and to promote research with and not on or about or for individuals and communities.

The Investigators go on to indicate that the Reddit's user base will facilitate recruitment which may work well, but how will those that are recruited be engaged in the various processes and not just provide data for the Investigators. The Investigators went on to say that they will have public contributors involved in the dissemination of findings; however, the young men that participated in the various aspects of the study should be the ones that disseminate the findings as they really own the data and are sharing it with the Investigators. Have the quantitative tools being used been validated for young men? Maybe the quantitative analysis has meaning but given that this is a convergent mixed-methods participatory study, it would seem that there might be various ways to help the young men understand the results of their contributions (quantitative and qualitative) and to further participate in next steps given that this protocol is for a pilot study. All aspects of qualitative rigour need to be addressed not just Feasibility Indicators (quantitative) or Triangulation.

I would encourage you to ensure that all aspects of qualitative rigour need to be addressed: credibility, confirmability, dependability and transferability along with member-checking and spending time in the community. These all contribute to the success of any qualitative study.

Having facilitated similar work in the past, relationships need to be developed with the young men being invited to participate in the project before any questions are asked. The questions being asked by Investigators are not necessarily the important questions to the young men maybe there are others e.g. safety.

VERSION 1 - AUTHOR RESPONSE

**Reviewer comment**	**Response**
Mixed-methods integration unclear	We clarified the distinct but complementary purposes of the qualitative and quantitative strands and added a dedicated subsection explaining how findings will be analysed separately and integrated during interpretation through triangulation and joint displays.

Purpose of qualitative vs quantitative methods unclear	We clarified that the qualitative component explores young men's perspectives on barriers, facilitators, and preferred approaches to engagement in mental health research, while the quantitative component evaluates the feasibility, acceptability, and potential influence of the combined photovoice–AI process on empowerment and engagement-related constructs.
Participatory framing insufficiently developed	We expanded the manuscript to clarify how lived experience perspectives, including input from an expert by experience informed the study design, recruitment approach, workshop structure, and discussion prompts, and how participants shape the direction of discussions during the AI process.
Concern that participants may only provide data rather than co-create the project	We clarified that the photovoice training session functions as an initial relational and collaborative space, that participants contribute to interpretation and dissemination processes, and that opportunities for co-authorship and dissemination involvement will be offered.
Relationship-building prior to data collection not sufficiently addressed	We revised the description of the photovoice training session to emphasise relational engagement, collaborative discussion, and the establishment of a psychologically safe environment prior to formal data generation.
Questions may reflect researcher assumptions rather than participant priorities	We clarified that workshop discussions are flexible and participant-led, and that participants are encouraged to identify issues they themselves consider important regarding engagement in mental health research.
Dissemination should involve participating young men	We expanded the dissemination section to state that participants will be invited to contribute to dissemination events, interpretation discussions, and potential co-authored outputs relating to their experiences of the participatory process.
Qualitative rigour insufficiently articulated	We strengthened the qualitative analysis section to explicitly address credibility, dependability, confirmability, transferability, reflexivity, triangulation, and member reflections.
Concern regarding participatory authenticity/community engagement	We clarified that this is a participatory-informed pilot study focused on collaboratively developing and testing engagement methods, rather than a full community-based participatory research design
Quantitative tools and meaning for young men unclear	We clarified that quantitative measures are exploratory and are included to assess feasibility, acceptability, and sensitivity to

	change within this pilot study. Validated measures were selected to explore whether participants felt empowered to be involved in such activities.

In addition, we have replaced “LEPs” with “participants” to avoid confusion arising from the concurrent use of the term “participant,” and we have redesigned the figure to present the same information in a clearer format.
